# Practical Microscopy—A Course of Normal Histology for Students and Practitioners of Medicine

**Published:** 1888-02

**Authors:** 


**Practical Microscopy—A Course of Normal Histology for Students and Practitioners of Medicine.** By MAURICE N. MILLER, M. D., Director of the Department of Normal Histology in the Loomis Laboratory, University of City of New York. New York: Wm. Wood & Co. 1887.

This book we would commend most highly. There is no want of excellent books on the same subject, but, as a rule, they are adapted for the advanced student and skilled microscopist. The majority of doctors who have microscopes, however, have not had the advantages of modern laboratory training, and to them a work which gives the processes for the preparation and exhibition of tissues, which are simple and practical and free from details of technique applicable only to the expert, will be of great service. Again, the work differs from many more pretentious works, in that the illustrations, which are numerous, are mostly original, being from the author's pen drawings. All of them are good, and some of them exceptionally beautiful and true. Those of our readers who do any serious work with the microscope, should buy this book.

